# Antifungal and Antiparasitic Drug Delivery

**DOI:** 10.3390/pharmaceutics12040324

**Published:** 2020-04-04

**Authors:** Juan José Torrado, Dolores R. Serrano, Javier Capilla

**Affiliations:** 1 Departament of Pharmaceutics and Food Technology, School of Pharmacy, Complutense University of Madrid, 28040 Madrid, Spain; 2 Unitat de Microbiologia, Facultat de Medicina i Ciències de la Salut, Universitat Rovira i Virgili and Institut d’Investigació Sanitària Pere Virgili (IISPV), 43201 Reus, Spain; javier.capilla@urv.cat

**Keywords:** liposomes, transferosomes, nanoparticles, emulsions, candidiasis, aspergillosis, azoles, amphotericin B, combined therapy, quality by design, leishmaniasis, malaria, trypanosomiasis

## Abstract

Fungal and parasitic diseases affect more than a billion people across the globe, one-sixth of the world’s population, mostly located in developing countries. The lack of effective and safer treatments combined with a deficient diagnosis lead to serious chronic illness or even death. There is a mismatch between the rate of drug resistance and the development of new medicines. Formulation of antifungal and antiparasitic drugs adapted to different administration routes is challenging, bearing in mind their poor water solubility, which limits their bioavailability and efficacy. Hence, there is an unmet clinical need to develop vaccines and novel formulations and drug delivery strategies that can improve the bioavailability and therapeutic effect by enhancing their dissolution, increasing their chemical potency, stabilising the drug and targeting high concentration of drug to the infection sites. This Editorial regards the ten research contributions presented in the Special Issue “Antifungal and Antiparasitic Drug Delivery”.

In order to obtain new antifungal and antiparasitic drug delivery systems, scientists of different disciplines have to collaborate in coordinated research teams. This volume includes ten papers, five of them about antifungal formulations and other five related to antiparasitic formulations. Amongst fungal infections, candidiasis has received special attention due to its world prevalence, as well as leishmaniasis as a parasitic disease. Interestingly, an old molecule, amphotericin B, is the active component studied in six out of the ten papers. Other active components also studied are butenafine, praziquantel, fluconazole, meglumine antimoniate and the enolase-base vaccine.

The administration route plays a key role in the development of novel antifungal and antiparasitic formulations. In this issue, a special focus on oral, parenteral and topical formulations is highlighted. Dosage forms are obviously related to the administration route. For example, suspensions, solutions and tablets are developed for oral administration while semisolid gels and wound patches are fabricated for topical application. Different parenteral administration routes are covered, such as subcutaneous, intravenous and at the bone cavity.

The originality of the new formulations proposed are usually based on the selection of already approved excipients along with the active components, such as Montanide™ Petgel A as vaccine adjuvant [1], poly(vinyl alcohol) [2], Poloxamer 407™ [3], a combination of Capryol 90™, Peceol™ and Labrasol™ [4], dextran and maltodextrin [5], a combination of modified chitosan nanoparticles with a standardized extract of cultured *Lentinula edodes* mycelia (AHCC™) [6], ground calcium carbonate [7], poly (d,l-lactide-*co*-glycolide) 50:50 [8] and Sepigel 305™ [9]. Only in one paper [10], authors have synthesized a new material based on copolymers of poly(ethyleneglycol) and poly(ɛ-caprolactone) conjugated with retinol as drug vehicle. Moreover, the characterization of the new formulations is described in detail in the ten papers with a special focus on toxicity and efficacy studies required in order to bring to the market these formulations. This Special Issue is an update on novel drug delivery strategies of antifungal and antiparasitic drugs to treat both topical and systemic infections. A brief description of the ten research papers included in the issue is described below.

Tellez-Martínez et al. propose a new vaccine based on recombinant enolase-Montanide™ PetGel A against virulent fungus *Sporothrix schenckii*. The incorporation of Montanide™ PetGel A as adjuvant was able to induce specific Th1 response and protective immunity against the fungal in Balb/c mice [1]. Interestingly, the virulence of *S. schenckii* was enhanced by toluene exposure. Toluene is an example of environmental contaminant. In this work, authors proved that the combination of some environmental contaminants can enhance the virulence of pathogen agents. Effective vaccines are an important pharmacological tool to protect us against this type of severe infections.

The work of Alexandrino-Junior et al. is a clear example of the potential pharmacological effect of new formulations of old drugs. Amphotericin B was formulated on a poly(vinyl-alcohol) hydrogel as a new topical formulation for the treatment of cutaneous leishmaniasis [2]. Although topical treatment of cutaneous diseases seems to be an ideal approach, conventional topical formulations of amphotericin possess low activity on cutaneous leishmaniasis due to permeability issues. Nevertheless, these new hydrogels developed in this work have exhibited, in vitro, a promising antiparasitic activity against *Leishmania* parasites and also against some fungal infections.

Sosa et al. performed an interesting study whose aim was the development and evaluation of a topical formulation of amphotericin B for the treatment of dermal and vaginal candidiasis [3]. Poloxamer 407™ was selected as excipient based on its thermoreversible properties. This excipient is liquid at low temperatures (4–5 °C) but turns into a semisolid gel above 32 °C. A thermoreversible gel containing amphotericin was developed and evaluated. Ex vivo permeation studies on human skin and pig vaginal mucosa showed that no permeation was observed. In vitro, antifungal activity studies against *Candida* spp showed that this formulation was more efficient than free amphotericin. Moreover, the amount of amphotericin remaining on the skin and vaginal mucosa was high enough to obtain antifungal activity.

Bezerra-Sousa et al. described the preparation of an oral nanomedicine of butenafine for visceral leishmaniasis [4]. Butenafine is currently used as a topical antifungal drug with low oral bioavailability. In this work, the low solubility of butenafine was increased by preparation of optimized self-nanoemulsifying drug delivery systems which have proved in vitro to be effective against promastigotes and amastigotes of *Leishmania infantum*. Moreover, these promising systems were then transformed by spray-drying into a solid dosage form of butenafine. Development of solid oral nanomedicines enables the non-invasive and safe drug administration, being a cost-effective and readily scalable repurposed medicine for visceral leishmaniasis. 

Serrano et al.’s work focused on the design of fast-dissolving orodispersible films of amphotericin B for oropharyngeal candidiasis [5]. Amphotericin B is a low water soluble antifungal drug. A quality-by-design study was applied to select the best combination of GRAS excipients. A fast disintegration film with quick amphotericin release in artificial saliva and high in vitro efficacy against several *Candida* spp. was obtained.

Pérez-Cantero et al. carry out an interesting study related to the increased prophylactic efficacy of parenteral and oral amphotericin B treatments against aspergillosis when combined with standardized extract of cultured *Lentinula edodes* mycelia (AHCC™) [6]. Amphotericin was encapsulated in modified chitosan-nanoparticles suitable for oral administration. The addition of AHCC™ significantly improved the efficacy of both oral and parenteral treatments in a mice model of experimental aspergillosis. Moreover, the weight loss of treated animals was lower when AHCC™ was administered, suggesting a protective effect of the extract. In relation to the control group, treated animals showed stimulation of the Th1 immune response, which can explain the improvement of its efficacy.

The work of Borrego-Sánchez et al. focused on the increase of solubility and dissolution rate of praziquantel [7]. Praziquantel is also a poorly water-soluble antiparasitic drug, highly effective against schistosomiasis. Ground calcium carbonate is a cheap, hydrophilic porous carrier that was combined with praziquantel by using two easily scalable processes: physical mixture or solid dispersions. An in vitro dissolution test proved that solid dispersions increase drug solubility and dissolution rate. In vitro cytotoxicity studies against HTC116 cells showed that the praziquantel solid dispersions are safe.

Hsu et al. studied how amphotericin B and fluconazole can be incorporated into resorbable beads [8]. These beads are made of biodegradable Poly(d,l-lactide-*co*-glycolide) (50:50) and they were fabricated using a compression-molding method. The beads were evaluated, showing that the in vitro release of the fluconazole beads was better than the one obtained from amphotericin B beads. The in vivo assay in rabbits showed a sustained antifungal activity of fuconazole for more than 49 days, and thus, was suitable for the treatment of bone infections.

Berenguer et al. developed and characterized a semi-solid gel dosage form of meglumine antimoniate for the topical treatment of cutaneous leishmaniasis [9]. The gel is easy to prepare and its main excipient is Sepigel 305™. It was stable for over 6 months. The pH and rheological characteristics were suitable for topical application. Ex vivo permeation studies in human skin show low permeation and high retention in the skin layer, so low systemic toxicity and enhanced local activity can be expected from this formulation. Low toxicity and good tolerance were observed in keratinocyte cell lines and human volunteers, respectively. In vitro anti-leishmanial activity of the gel showed a reduction of the IC_50_ compared to the reference solution. This new formulation could be a promising alternative for topical treatment of cutaneous leishmaniasis.

Rodriguez et al. described the development of amphotericin B micellar formulations based on copolymers of poly(ethyleneglycol) and poly(ɛ-caprolactone) conjugated with retinol [10]. Biodegradable and biocompatible polymers were initially synthesized and then conjugated with retinol. These micellar formulations were less haemolytic than Fungizone™. Furthermore, the antifungal activity of amphotericin incorporated in these new formulations showed a reduction of the MIC of up to eight-fold compared with reference Fungizone™. The low toxicity and high in vitro antifungal activity of these formulations make them good candidates for future in vivo experiments.
